# Nanoparticle ultrastructure allows reversible pH sensitivity using ^19^F NMR and *in vivo*^19^F MRI

**DOI:** 10.1039/d5na01005e

**Published:** 2026-01-28

**Authors:** Alvja Mali, Mariah Daal, Natalia Jirát-Ziółkowska, Nicolas Stumpe, Naiara Larreina Vicente, N. Koen van Riessen, Visakh V. S. Pillai, Francesco Simone Ruggeri, Cyril Cadiou, Françoise Chuburu, Daniel Jirak, Paul B. White, Mangala Srinivas

**Affiliations:** a Department of Cell Biology and Immunology, Wageningen University and Research P. O. Box 338 6700 AH Wageningen The Netherlands mangala.srinivas@wur.nl; b Institute for Clinical and Experimental Medicine Prague Czech Republic; c Institute of Biophysics and Informatics, First Faculty of Medicine, Charles University Prague Czech Republic; d Institute for Molecular Cardiology, Heinrich Heine University Düsseldorf Germany; e Physical Chemistry and Soft Matter, Wageningen University and Research Wageningen The Netherlands; f University of Reims Champagne Ardenne, CNRS, ICMR UMR 7312 Reims France; g Faculty of Health Studies, Technical University of Liberec Liberec Czech Republic; h Institute for Molecules and Materials, Radboud University Nijmegen The Netherlands; i Cenya Imaging B.V. Amsterdam The Netherlands

## Abstract

Fluorine-19 magnetic resonance imaging (^19^F MRI) is a powerful imaging modality that provides background-free imaging. Its sensitivity can be modulated through environmentally responsive probe designs exploiting paramagnetic relaxation enhancement (PRE) and related mechanisms. Here, we present a novel pH-sensitive ^19^F MRI nanosystem in which signal activation is governed by nanoparticle (NP) ultrastructure. Poly(lactic-*co*-glycolic acid) (PLGA) NPs were synthesized co-encapsulating perfluoro-15-crown-5-ether (PFCE) and lipophilic gadolinium (Gd) chelates, yielding either single-core or multi-core structures. While single-core NPs, the conventional structure for polymer-encapsulated perfluorocarbons, remained pH-insensitive, multi-core NPs exhibited a pronounced and reversible pH-dependent ^19^F signal modulation. The ^19^F *T*_1_ decreased slightly (from ∼700 to ∼600 ms) in both NP types due to the presence of Gd, whereas *T*_2_ shortened markedly at neutral pH (∼20 ms, “OFF” state) and increased substantially under acidic conditions (“ON” state); this variation was absent in single-core NPs. The pH-dependent ^19^F *T*_2_ behaviour was consistent across both high-field (14 T) and low-field (3 T) MRI. Such behaviour of multi-core NPs was validated *in vitro* and *in vivo*, as the ^19^F signal reappeared only after lysosomal internalization in RAW 264.7 cells and was selectively activated within acidic tumour regions, but not at the neutral subcutaneous injection site. By exploiting nanoparticle ultrastructure to achieve reversible signal switching, this work introduces a general strategy for activatable ^19^F MRI probes for imaging applications in tumours and other pH-sensitive disease environments. It demonstrates, for the first time, PRE-driven signal enhancement and suppression governed by structural rearrangement rather than chemical modification in response to pH variation.

## Abstract 2

PLGA nanoparticle internal structure enables pH-responsive fluorine-19 magnetic resonance imaging (^19^F MRI) upon co-encapsulation of perfluorocarbon and gadolinium.

## Graphical abstract

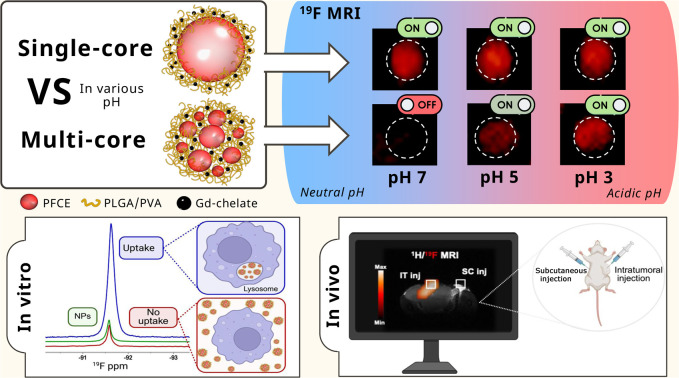

## 1 Introduction

Non-invasive imaging techniques are crucial for early disease detection, driving the need for more sensitive and specific molecular imaging probes. Among emerging strategies, nanoparticle (NP)-based contrast agents have gained particular attention for their ability to enhance imaging sensitivity, improve targeting, and support multimodal imaging. Their tunable physicochemical properties, such as size, surface charge, and composition, enable control over circulation time, tissue accumulation, and contrast agent encapsulation, making them adaptable tools for a wide range of imaging modalities.^1,2^ These advantages have made NPs increasingly relevant in biomedical applications aimed at visualizing molecular and cellular processes *in vivo*.^3^ Clinical imaging enables real-time, non-invasive monitoring of physiological and pathological conditions, with magnetic resonance imaging (MRI) offering superior soft-tissue contrast, deep tissue penetration, and high spatial resolution without ionizing radiation.^4^

However, conventional proton (^1^H) MRI suffers from strong background signals caused by the high-water content in biological tissues (65–90%), reducing contrast and making it difficult to distinguish specific targets, particularly early-stage tumours.^4–6^ This has led to growing interest in alternative MRI-active nuclei, such as ^23^Na,^7^^19^F,^8,9^ and ^31^P,^10,11^ which offer improved specificity and signal clarity. Among them, fluorine-based MRI (^19^F MRI) has gained significant attention due to its high sensitivity, comparable to that of protons, and its exceptional specificity, as fluorine is nearly absent in biological tissues (≤10^−6^ M).^12,13^ These characteristics have made ^19^F MRI a powerful tool for diverse applications, including tracking cellular therapies and quantitative imaging.^14–17^ Among the different fluorine labels, perfluorocarbons (PFCs) are the most commonly used in ^19^F MRI, owing to their high fluorine content, excellent molecular mobility, and favourable safety profile.^18^ PFC-based agents have also been used clinically.^19,20^

Beyond direct fluorine imaging, activatable ^19^F MRI probes enhance sensitivity by exploiting changes in relaxivity^6,21^ or chemical shift^22,23^ to detect biological processes or trigger drug release.^24,25^ Stimulus-induced relaxivity alterations can create on/off signal switches, significantly improving ^19^F MRI sensitivity. These effects are primarily regulated by their structural properties, particularly their influence on transverse relaxation (*T*_2_).^26^ Two key strategies regulate ^19^F MR signal modulation: (1) fluorine nuclei tethered to paramagnetic centres *via* stimuli-responsive linkers, where proximity modulates the paramagnetic relaxation enhancement (PRE) effect,^27–29^ and (2) reversible fluorine agent aggregation, where restricted motion quenches the signal and disassembly restores it.^21,30,31^ PRE is highly distance-dependent, following an inverse sixth power relationship (*r*^−6^) with fluorine–chelate separation.^32–34^ Thus, precise nanoscale control over molecular reconfigurations is essential for responsive probe design.^26,35^ Nanomaterials provide ideal platforms for designing such activatable probes, as their tuneable physicochemical properties allow optimization of PRE effects and fluorine relaxation behaviour for enhanced responsiveness.^36,37^

Among external stimuli, pH is particularly relevant, as it is tightly regulated in normal tissues (7.2–7.4) but disrupted under pathological conditions such as inflammation and cancer.^38^ Tumours exhibit a characteristic acidic extracellular microenvironment due to metabolic reprogramming.^39,40^ As a hallmark of disease, these variations make pH-sensitive imaging valuable for detection and monitoring.^41^

Most pH-responsive polymer NPs used for ^19^F MRI contain ionizable groups, such as tertiary amines, with varying acid dissociation constants (p*K*_a_ values).^42–46^ Fluctuations in pH induce protonation or deprotonation of these groups, leading to polymer swelling or shrinking, which indirectly modulates^47^ the fluorine signal.^48,49^ However, a key challenge for these agents remains increasing the usable fluorine signal while maintaining sufficient fluorine mobility.^45^ Higher fluorine content often increases hydrophobicity and fluorophilicity, leading to aggregation in aqueous environments, which reduces molecular mobility and signal intensity, and may cause toxicity risks *in vivo*.^18,24^

Our previous research demonstrated the successful co-encapsulation of lipophilic gadolinium (Gd) chelates and perfluoro-15-crown-5-ether (PFCE) within a polymer matrix (poly(d,l-lactide-*co*-glycolide), PLGA), yielding NPs with either multi-core or single-core structures, as confirmed by Small-Angle Neutron Scattering (SANS).^50–52^

While PRE in PFCs typically requires fluorophilic chelators,^53,54^ in our earlier methodological study,^51^ we showed that NP ultrastructure alone significantly influences ^19^F MR relaxation, with multi-core NPs exhibiting stronger paramagnetic effects than single-core NPs. That same work also revealed only preliminary indications of pH-sensitive ^19^F MR relaxation, uniquely associated with the multi-core configuration, without systematic mechanistic analysis or validation under physiologically relevant conditions. Moreover, the internal structure was previously shown to influence *in vivo* clearance^52^ and *in vitro* cellular uptake.^55^

Building on these findings, the present study systematically investigates the mechanism, reversibility, and physiological relevance of this pH responsiveness. Using both high- and low-field MRI (14 T and 3 T), we observed pH-dependent ^19^F MRI signal changes, with multi-core NPs exhibiting pronounced pH responsiveness even under physiologically relevant conditions. In proof-of-concept *in vivo* experiments (7 T MR), a ^19^F signal was observed only in the tumour and not subcutaneously. Overall, we have built on earlier structural observations towards a translational imaging context, with further mechanistic understanding of the reversibility of the signal generation.

## 2 Results and discussion

**Fig. 1:**
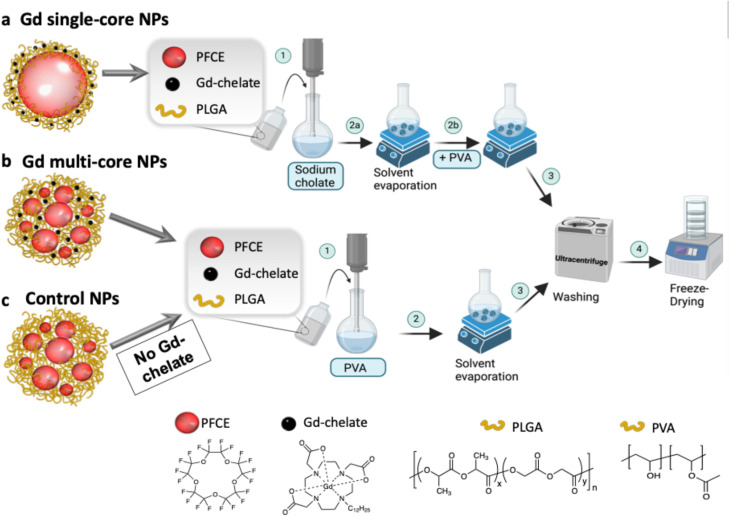
Schematic representation of PLGA–PFCE NP formulation using a single oil-in-water emulsion process for (a) Gd single-core, (b) Gd multi-core, and (c) control NPs. (a) Gd single-core NPs are prepared by sonication of the organic phase with sodium cholate as a surfactant (1), which is later replaced by PVA after solvent evaporation (2a and 2b). (b) Gd multi-core and (c) control NPs are directly formulated using PVA as the surfactant (1), with control NPs omitting the Gd-chelate in the organic phase. After solvent evaporation (2), all NPs are washed to remove excess PVA and free Gd-chelate (3), collected by ultracentrifugation, and freeze-dried (4). Chemical structures of key components, PFCE, Gd-chelate, PLGA, and PVA, are shown at the bottom of the figure for reference.

PFCs are advantageous for ^19^F MRI due to their high fluorine content but present challenges such as fluorophilicity, low surface tension, and slow clearance.^18^ Customizable nanocarriers, such as lipid- or polymer-based NPs, address these limitations.^18,56–58^ Our group has focused on optimizing and characterizing PLGA-loaded PFCE NPs, demonstrating that slight procedural modifications yield NPs with either multi-core or single-core ultrastructure (Fig. 1a and b).^50,59^ The ultrastructure plays a crucial role in PFCE clearance, with multi-core NPs exhibiting 15-fold faster clearance *in vivo* compared to single-core NPs.^52^ These structural differences also influenced MR properties, particularly when a lipophilic Gd-chelate was co-encapsulated with PFCE in the PLGA matrix. Gd entrapment increased with the amount of Gd-chelate added, with higher encapsulation observed for chelates containing longer hydrophobic chains.^51^ However, the consistent pH sensitivity across different Gd chelates suggests that the NP ultrastructure, rather than the chelator itself, determines the response. Based on these findings, only the chelator with the longer hydrophobic tail was used because of its slightly higher encapsulation efficiency.^51^ In this study, three types of NPs were investigated: (1) Gd single-core, (2) Gd multi-core, and (3) control NPs (Gd-free) (Fig. 1a–c). Gd single-core and multi-core NPs were used to investigate the role of NP ultrastructure in pH sensitivity, while control NPs served to determine whether the observed effects were intrinsic to the PLGA matrix or specifically due to the co-encapsulation of Gd-chelate and PFCE.

To ensure comparability across pH sensitivity experiments, NPs were standardized based on (1) Gd entrapment measured by ICP-OES, (2) PFCE entrapment (% wt), and (3) *T*_1_ and *T*_2_ relaxation times measured by ^19^F NMR (Table 1). Due to inherent batch-to-batch variation, Table 1 reports average values from multiple batches used in this study. While Gd entrapment remained consistent across NP types, PFCE entrapment varied slightly, with Gd multi-core NPs exhibiting the lowest encapsulation efficiency. The co-encapsulation of Gd-chelate did not significantly alter the NP parameters, such as diameter and PDI (Table S1), with multi-core NPs (∼200 nm, PDI 0.2) and single-core NPs (∼160 nm, PDI 0.15) showing similar size distributions.

**Table 1:** Characterization of Gd multi-core, Gd single-core, and control NPs. The table presents average values for: (1) Gd entrapment (µg of Gd per mg of NPs) determined by ICP-OES; (2) *T*_1_ and *T*_2_ relaxation times (ms) measured *via* 500 MHz NMR in water (pH 7); and (3) PFCE entrapment (% wt). Data represent averages from 9, 4, and 5 batches, respectively

NP type	Batches	Gd entrapped µg (Gd) per mg (NP)	^19^F *T*_1_ (ms)	^19^F *T*_2_ (ms)	PFCE% wt
Gd multi-core	9	2.3 ± 0.7	550 ± 40	21 ± 9	14 ± 2
Gd single-core	4	2.0 ± 1.0	640 ± 15	55 ± 10	18 ± 4
Control	5	N/A	750 ± 35	390 ± 50	22 ± 4

The encapsulation of paramagnetic chelates significantly affected ^19^F MR properties, particularly in multi-core NPs, where it induced line broadening, potentially affecting quantification accuracy. The paramagnetic effect was more pronounced in *T*_2_ than in *T*_1_, with greater *T*_2_ shortening observed in multi-core NPs due to *T*_2_'s higher sensitivity to local magnetic field variations, which are amplified by paramagnetic agents such as Gd.^32,60^ The effect of paramagnetic ions on the relaxation times of ^19^F nuclei depends on their distance from the fluorinated core.^54,61,62^ Previous SANS measurements indicated that each PFCE core in multi-core NPs has a radius of 9–12 nm with a 4 nm shell thickness, while single-core NPs have a core radius of approximately 20 nm with a similar shell thickness.^50^ This configuration results in a maximum Gd to PFCE distance of approximately 15 nm in multi-core NPs, compared to about 24 nm in single-core NPs.^51^ Given that PRE decreases steeply with increasing distance,^35^ the robust PRE effect observed in multi-core NPs suggests that additional structural factors, such as PFCE confinement or restricted mobility, may enhance fluorine relaxation beyond what would be expected based on spatial averages alone. In contrast, the larger, less confined single-core NPs allow for greater PFCE mobility, reducing these interactions and weakening PRE. These structural differences likely account for the stronger PRE effect observed in multi-core NPs.

**Fig. 2:**
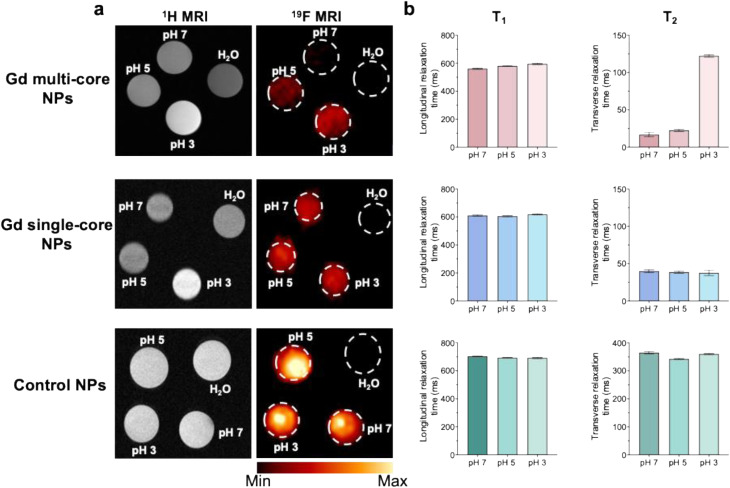
(a) ^1^H and ^19^F MRI (14 T) of Gd multi-core, Gd single-core, and control NPs (concentration of 6.5 mg mL^−1^) at pH 7, 5, and 3, with water (H_2_O) as a reference. (b) Corresponding ^19^F *T*_1_ (longitudinal) and *T*_2_ (transverse) relaxation times under the same pH conditions. Relaxation values are voxel-averaged from a single imaging run with one NP batch.

^19^F PRE has long been used to improve ^19^F MR signal sensitivity and to develop stimuli-responsive systems by incorporating specific functional groups sensitive to environmental changes, which modulate the ^19^F signal.^18,24,26,28^ Beyond chemical modifications, the internal structure of PLGA NPs also influences PRE upon pH response. Indeed, Gd single-core NPs showed minimal changes in ^19^F relaxation times across different pH conditions. In contrast, multi-core NPs exhibited a pronounced response to pH variations, with *T*_2_ relaxation times increasing 15-fold from neutral to acidic pH.^51^^19^F MRI at 14 T (Fig. 2a) confirmed these observations, showing a marked ^19^F signal increase for multi-core NPs under acidic conditions, whereas no changes were observed in single-core and control NPs. The *T*_2_ relaxation time of multi-core NPs increased with decreasing pH, while the *T*_1_ and *T*_2_ values for control NPs and Gd single-core remained stable (Fig. 2b and S1). Notably, even at acidic pH, the *T*_2_ of Gd multi-core NPs does not fully recover to the level observed for pristine PFCE NPs. This likely reflects residual PRE effects from the encapsulated Gd chelate, which continues to influence nearby ^19^F nuclei.

In addition, control NPs exhibited higher ^19^F signal intensity compared to Gd-loaded NPs, attributed to their higher PFCE content (% wt) (Table 1). Additionally, the defined area of higher intensity (Fig. 2a) likely reflects NP sedimentation due to the shape of the sample tube.

While ^19^F PRE effects are primarily dictated by the proximity and interaction of Gd chelate with the fluorinated core, the enhanced ^1^H MRI contrast observed at pH 3 for Gd multi-core NPs (Fig. 2a) suggests that acidic environments induce structural changes in the polymer matrix, increasing water permeability and Gd–water interactions. The ^1^H *T*_1_ relaxation time (Table S2 and Fig. S2) further supports this, showing a significant reduction in *T*_1_ at pH 3, while *T*_1_ values at pH 5 and 7 remain comparable. These findings suggest that acidic pH induces polymer swelling, likely due to carboxylic group protonation, which reduces rigidity and enhances water permeability, ultimately facilitating water access to Gd and amplifying its paramagnetic effects. Previous NMR spectroscopy studies corroborate this, confirming water's presence within the polymer network and its proximity to the PFCE cores.^50^

**Fig. 3:**
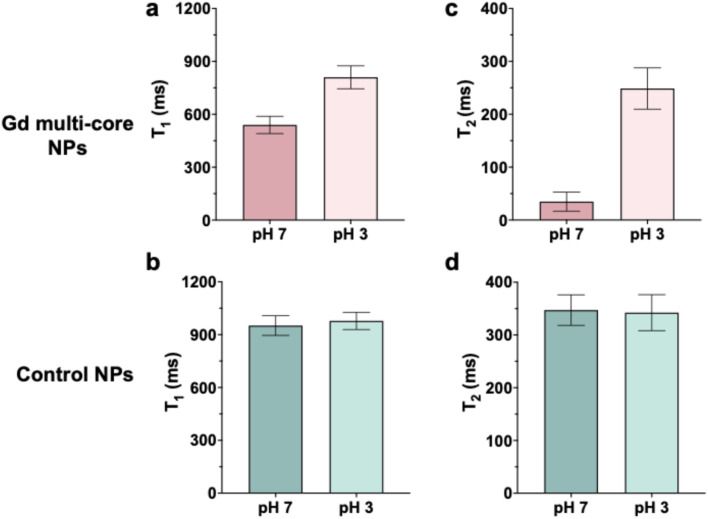
^19^F *T*_1_ (a and b) and *T*_2_ (c and d) relaxation times of Gd multi-core and control NPs. All samples were measured at the same concentration 6.5 mg mL^−1^ on a 3 T MR scanner.

To confirm the pH sensitivity observed at 14 T, multi-core NPs were further investigated using a low-field (3 T) MRI scanner (Fig. 3). The results at 3 T were consistent with those at 14 T, as *T*_1_ and *T*_2_ of control NPs remained constant across pH conditions (Fig. 3b and d). In contrast, multi-core NPs exhibited an increase in *T*_2_ under acidic conditions (Fig. 3c), corresponding to a brighter ^19^F signal and reinforcing their pH responsiveness.

Given the lack of pH sensitivity in single-core NPs, subsequent investigations focused on multi-core NPs with Gd-free PLGA–PFCE NPs used as controls to confirm the effect of Gd-chelate interactions rather than intrinsic polymer properties.

To rule out the possibility that the observed effects were due to PLGA hydrolysis or Gd-chelate release under acidic conditions, the stability of encapsulated Gd was previously confirmed using ^1^H NMR.^51^ Furthermore, Gd release was mimicked by supplementing GdCl_3_ (gadolinium chloride) to Gd-free NPs; while proton relaxation was similarly affected, the ^19^F signal remained unchanged, confirming that the lipophilic Gd-chelates remained confined within the hydrophobic PLGA matrix even in acidic environments.^51^

**Fig. 4:**
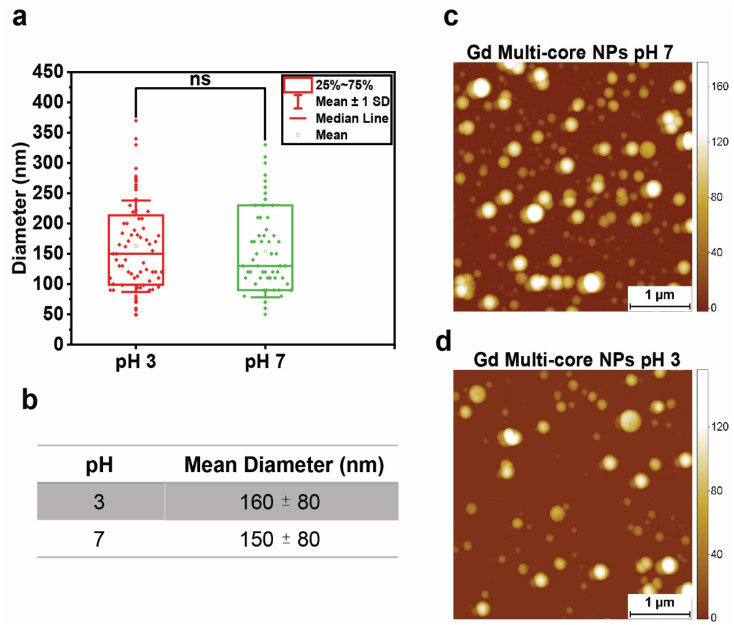
(a) Statistical analysis of the Gd multi-core NP diameter (nm) measured by AFM in acidic (pH 3) and neutral (pH 7) environments. Two-sample *t*-test for variance: *p* > 0.05. (b) Mean diameter of NPs under acidic and neutral pH conditions. (c and d) AFM images of Gd multi-core NPs at (a) neutral pH and (b) acidic pH. Scale bar: 1 µm.

With hydrolysis excluded, the pH-dependent effect was next examined in terms of possible NP swelling, which remained undetectable by DLS (Fig. S3 and Tables S3, S4). Therefore, the size of Gd multi-core NPs was measured using AFM at both acidic (pH 3) and neutral (pH 7) pH. While statistical analysis showed no significant size difference (Fig. 4a), the mean diameter at pH 3 was slightly larger (∼10 nm, Fig. 4b). Although this increase was not statistically significant, even minor changes in nanoscale dimensions could, in principle, influence the PRE effect due to its high sensitivity to minor distance variations in effective Gd–^19^F distance.^34^

Importantly, the absence of a statistically significant change in particle size indicates that geometric expansion alone is unlikely to account for the pronounced pH-dependent changes observed in ^19^F relaxation. Instead, these results suggest that additional mechanisms contribute, including pH-induced alterations in internal nanoparticle dynamics, such as increased PFCE mobility or changes in the effective correlation time within the polymer matrix, which can strongly modulate PRE without requiring large changes in average Gd–^19^F separation.

AFM imaging (Fig. 4c and d) further supported the structural stability of Gd multi-core NPs, showing that the NPs retained a spherical shape across different pH levels.

**Fig. 5:**
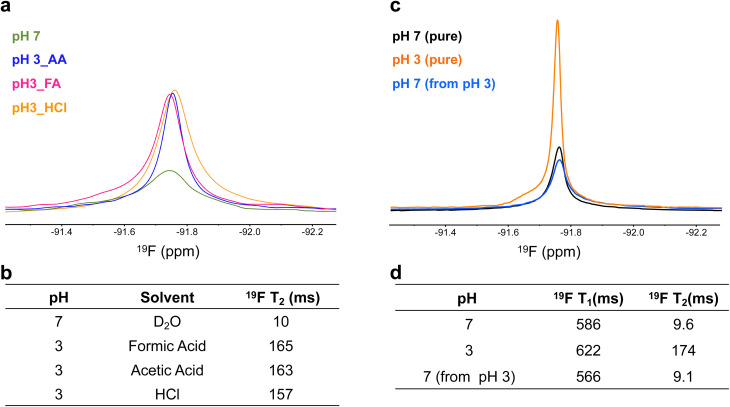
(a) ^19^F spectral line profiles of NPs resuspended in formic acid (FA), acetic acid (AA), hydrochloric acid (HCl), and deuterium oxide (D_2_O, pH 7) as a control, illustrating signal characteristics across different acidic environments. (b) ^19^F *T*_2_ relaxation times of NPs under the same conditions. (c) Reversible pH-dependent ^19^F signal modulation, showing the ^19^F signal of NPs resuspended at pH 7, pH 3 (HCl solution), and the same sample at pH 3 after neutralization to pH 7 with 5 N NaOH. (d) ^19^F *T*_1_ and *T*_2_ relaxation times corresponding to the same samples under the indicated pH conditions. All samples were measured by ^19^F NMR at 500 MHz with a concentration of 6.5 mg mL^−1^ for all conditions.

To determine whether this effect was driven solely by increased permeability and swelling or also influenced by the surrounding environment, ^19^F signal intensity and *T*_2_ values were examined in different acidic media. Gd multi-core NPs were resuspended in formic acid, acetic acid, HCl, and D_2_O. The consistent ^19^F signal intensities and *T*_2_ values (Fig. 5a and b) across these conditions suggest that the pH sensitivity is inherent to the NP structure rather than specific ion interactions. Furthermore, DLS measurements confirmed that the particle size remained stable, indicating that the NPs were not structurally degraded by the acid exposure (Table S3 and Fig. S3).

A key feature of the Gd multi-core NPs is their reversible pH-dependent ^19^F signal modulation, as observed by ^19^F NMR. To investigate this reversibility, samples from the same batch were compared: one resuspended at pH 7, one at pH 3, and the latter subsequently neutralized to pH 7 using 5 N NaOH. Initially, the ^19^F signal at pH 3 was higher, but upon neutralization, it decreased to match that of the sample originally resuspended at pH 7 (Fig. 5c). This behaviour aligns with the increase in *T*_2_ values under acidic conditions (Fig. 5d). Reproducibility of this reversible behaviour across independently prepared nanoparticle batches is shown in the SI (Table S5).

DLS measurements confirmed that the NPs remained structurally stable throughout the pH transition, with no effective changes in size or PDI (Table S4). A similar reversible effect was observed in NPs resuspended in acetic acid (Table S6), reinforcing that the pH responsiveness arises from the NP ultrastructure rather than the acidic medium itself.

To further investigate the pH responsiveness of Gd multi-core NPs under biologically relevant conditions, in-cell ^19^F NMR spectroscopy was performed using RAW 264.7 macrophages. Previous phantom experiments were conducted under non-physiological pH conditions, which do not fully reflect the moderate acidity of cellular compartments. Thus, assessing whether the NPs exhibit similar pH sensitivity under physiological conditions was necessary. For this purpose, cells were labelled with Gd multi-core and control NPs and analysed *via*^19^F NMR in a Shigemi tube containing culture medium with 5% D_2_O. Cell viability remained high before and after measurements.

**Fig. 6:**
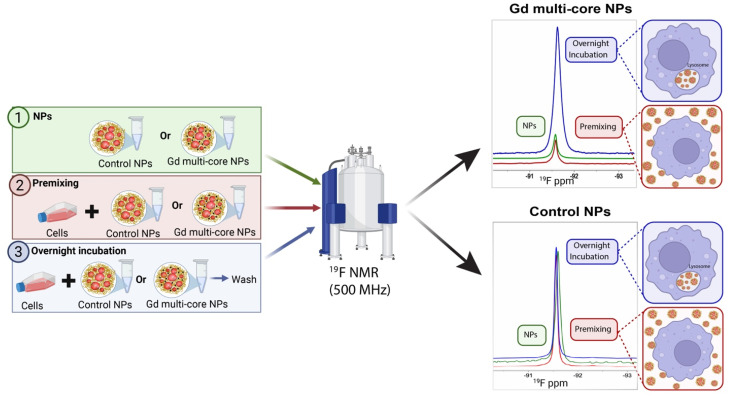
Experimental setup and ^19^F NMR spectra at 500 MHz for control NPs and Gd multi-core NPs with RAW 264.7 macrophages. The results present three conditions: premixing, overnight incubation, and NPs in medium (Biorender).

Three experimental conditions were analysed: (1) NPs in medium, to assess the influence of the medium's biochemical composition on pH responsiveness, (2) premixing, representing extracellular NP presence at pH 7, and (3) overnight incubation, mimicking NP uptake and intracellular processing (Fig. 6).

PLGA-based particles of this size are primarily internalized through fluid-phase pinocytosis and clathrin-mediated endocytosis.^63,64^ Due to their terminal carboxylic acid groups, negatively charged PLGA NPs tend to accumulate in lysosomes, unlike positively charged NPs, which can escape lysosomes and exhibit perinuclear localization.^65^ Therefore, we expected that the acidic lysosomal environment would influence the ^19^F relaxivity of the Gd multi-core NPs.

Under premixing conditions (Fig. 6), the ^19^F signal of Gd multi-core NPs was initially low, similar to the baseline signal of the NPs in medium. However, following overnight incubation, the ^19^F signal increased substantially, indicating responsiveness to the mildly acidic intracellular environment. In contrast, the control NPs exhibited consistently high signal intensity both before and after incubation, with no significant increase over time, indicating no pH responsiveness (Fig. 6). *T*_2_ values (Table S7) further support these observations, showing an increase for Gd multi-core NPs from ∼30 ms to ∼200 ms after incubation, whereas control NPs remained stable (∼750 ms). The fact that the *T*_2_ of Gd multi-core NPs remains at 208 ms, significantly lower than that of control NPs (Table S7), suggests that the ^19^F nuclei still experience the PRE effect from Gd. This indicates that Gd has not been entirely released, and the particles remain structurally intact, rather than undergoing complete degradation.

**Fig. 7:**
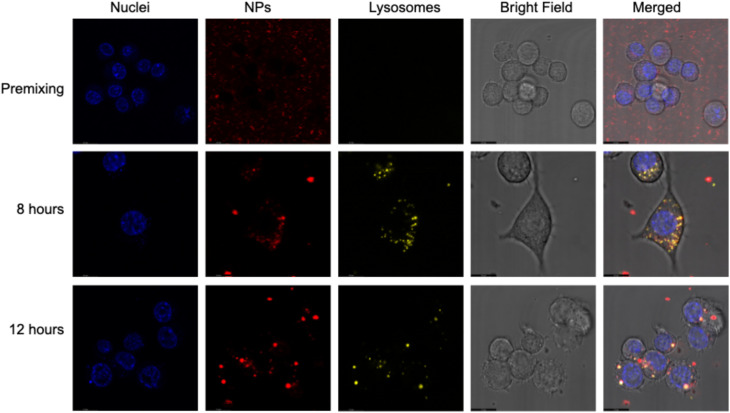
Confocal microscopy images of RAW 264.7 macrophages with NPs under three conditions: premixing and after 8 hours and overnight (12 hours) uptake. Nuclei are stained in blue (Hoechst EasyProbe), NPs in red (AttoOxa12), and lysosomes in yellow (LysoTracker™ Green DND-26). Live-cell imaging was performed using an inverted microscope with a 63× oil-immersion lens. Fluorescence and bright-field channels are displayed separately, along with the merged image. The signal from the NPs is visible in lysosomes after 8- and 12-hour incubation.

Confocal microscopy confirmed intracellular localization, showing that Gd multi-core NPs remained outside the cells under premixing conditions but colocalized with lysosomes after 8 hours and overnight incubation (12 hours) (Fig. 7). The increase in the ^19^F NMR signal after overnight incubation aligns with this lysosomal localization, suggesting that the mildly acidic environment enhances the pH responsiveness of Gd multi-core NPs under physiological conditions.

**Fig. 8:**
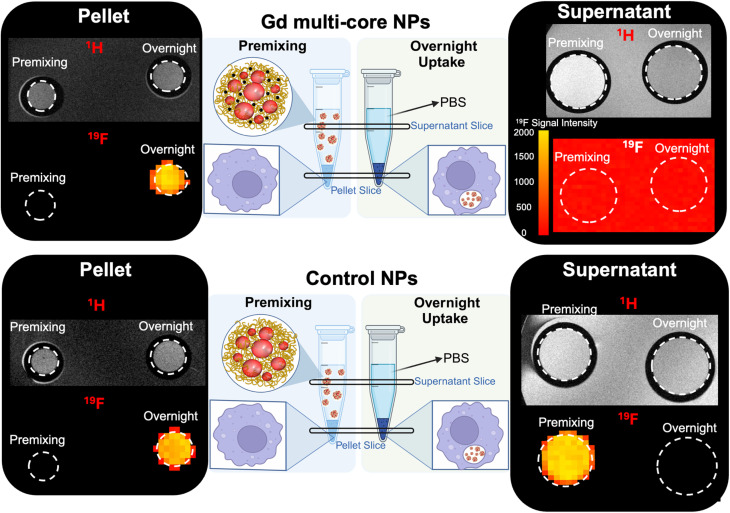
*In vitro* ^19^F and ^1^H MRI (14 T) of RAW 264.7 macrophages incubated with Gd multi-core and control NPs under premixing conditions and after overnight incubation. MR imaging was performed in two slices: one of the cell pellets (left) and one of the supernatants (right). To demonstrate the absence of a ^19^F signal in the supernatant for the Gd multi-core condition, the ^19^F signal is displayed without background suppression, showing only noise.

The trends observed in the NMR studies were further corroborated by *in vitro*^19^F and ^1^H MRI (14 T) of RAW 264.7 macrophages incubated with Gd multi-core or control NPs (Fig. 8). Cells were incubated under the same conditions described above for the in-cell NMR, namely, “premixing”, which mimics extracellular NP presence at neutral pH, and “overnight incubation”, which allows for intracellular processing in mildly acidic compartments. After incubation, cells were fixed, resuspended in PBS, and imaged. Both the pellet and the supernatant were imaged to distinguish signal contributions from internalized *versus* extracellular particles (Fig. 8).

In the supernatant of the cells incubated with Gd multi-core NPs, no ^19^F MR signal was detected after overnight incubation, as uninternalized NPs were washed away. Similarly, under the premixing conditions, the ^19^F signal remained undetectable, consistent with the previously observed “off” state due to the PRE effect of Gd (Fig. 8 and S4). However, under premixing condition, an increased ^1^H signal confirmed the presence of Gd multi-core NPs in the supernatant, indicating Gd-driven contrast enhancement (Fig. 8). This behaviour differs from that observed in aqueous suspension (Fig. 2), where ^1^H signal enhancement occurred only at pH 3, likely due to polymer swelling induced by carboxyl group protonation. In contrast, the enhanced ^1^H signal in PBS at pH 7 suggests that ionic interactions with salts in the buffer may also contribute to mild NP swelling. We hypothesize that these interactions disrupt polymer packing and increase hydration, allowing greater water permeability around the Gd chelates and enhancing relaxivity. However, this effect alone was insufficient to mitigate the PRE effect and restore the ^19^F MR signal. This indicates that while some structural rearrangement may occur in PBS, it does not significantly alter the Gd–PFCE proximity required to relieve PRE suppression.

In the cell pellet, no ^19^F MR signal was observed under the premixing conditions, consistent with the NPs remaining extracellular in PBS (pH 7), where the PRE effect continues to suppress the ^19^F signal. After overnight incubation, however, the ^19^F signal became clearly detectable in the pellet. This signal recovery suggests that intracellular uptake and subsequent trafficking into mildly acidic compartments, such as late endosomes or lysosomes, alleviated the PRE effect, likely through ultrastructural rearrangement that increased the average Gd–PFCE distance or altered PFCE mobility.

For the control NPs, which lack Gd, a clear ^19^F MR signal was observed in the supernatant under premixing condition and remained detectable in the pellet after overnight incubation (Fig. 8), with no evidence of signal modulation. This confirms that these NPs are not pH-responsive and that their signal remains stable over time regardless of location.

Interestingly, these findings reveal an additional advantage of Gd multi-core NPs for ^19^F MRI-based cell tracking.^66^ In *ex vivo* cell labelling workflows, it is often difficult to distinguish between truly internalized NPs and extracellular surface-bound particles that persist despite washing, potentially confounding *in vivo* imaging readouts. In our system, only particles that are internalized and processed within the mildly acidic intracellular environment undergo structural changes sufficient to restore the ^19^F signal. In contrast, extracellular particles remain undetectable by ^19^F MRI due to the ongoing PRE effect. This selective signal activation improves labelling specificity and offers a promising strategy to enhance the reliability of ^19^F MRI for tracking labelled cells *in vivo*.

**Fig. 9:**
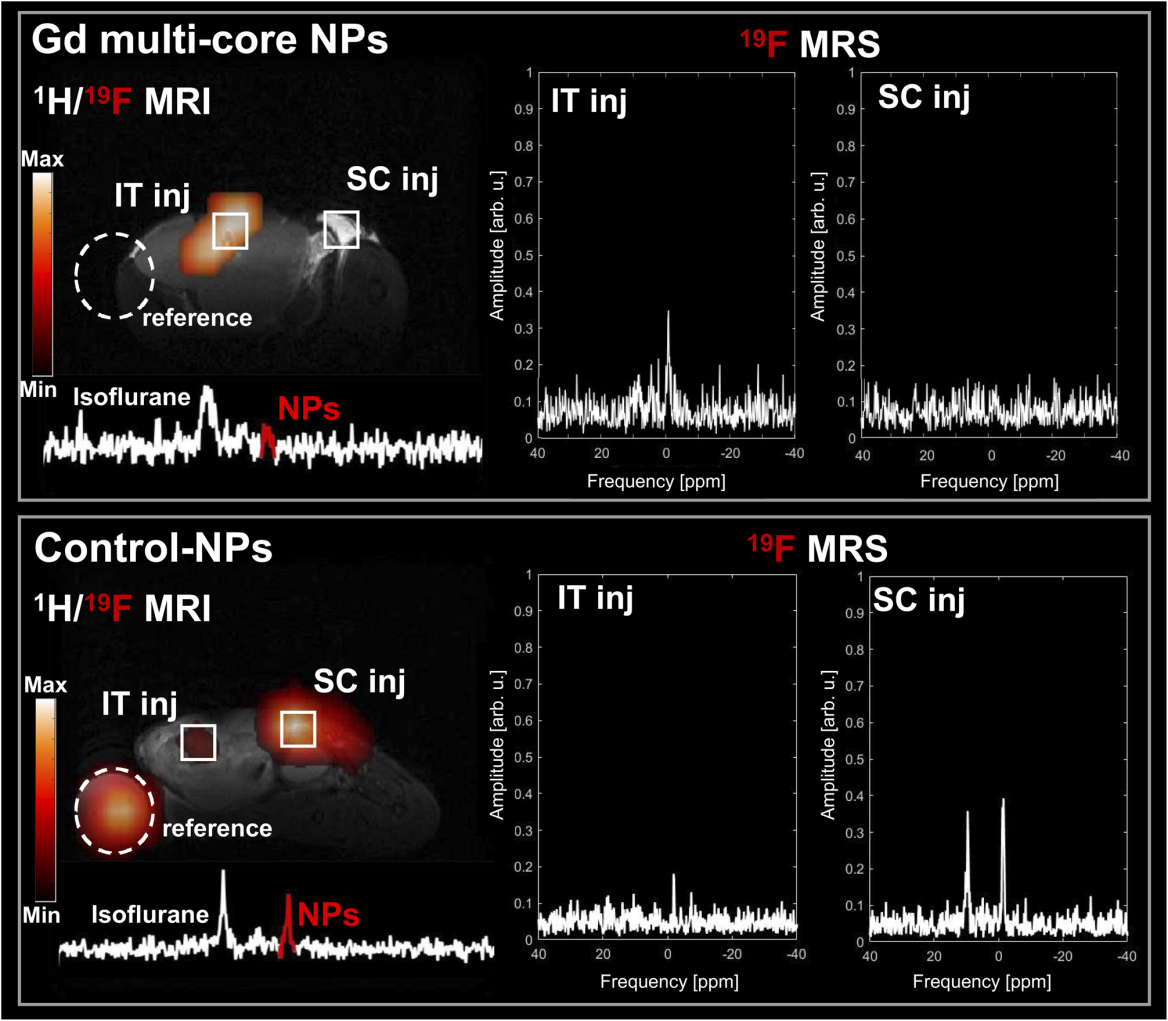
*In vivo* ^1^H and ^19^F MRI (7 T) of Gd multi-core and control NPs (5 mg/100 µL) following intratumoral (IT) and subcutaneous (SC) injections, with a reference (2 mg/100 µL) on site. On the left, merged ^1^H/^19^F MR images of mice, with corresponding non-localized ^19^F MR spectra from NPs, shifted from the isoflurane anaesthesia. On the right, ^19^F MR spectra from voxels within IT and SC injections. The fluorine signal is marked in red. Total ^19^F MRI acquisition time: 60 min.

Building on the strong *in vitro* pH response of Gd multi-core NPs, their behaviour was further evaluated in an *in vivo* tumour model as a proof of concept. As shown in Fig. 9, the Gd multi-core NPs produced a strong ^19^F MR signal within the tumour, whereas no detectable signal was observed in the reference tube or at the subcutaneous injection site, both mimicking the pH 7 conditions previously studied *in vitro*. The presence of the ^19^F MR signal within the tumour was further confirmed by *ex vivo* imaging after tumour extraction (Fig. S5), demonstrating the responsiveness of Gd multi-core NPs to the acidic tumour microenvironment. In contrast, control NPs remained clearly visible by ^19^F MR in both the subcutaneous injection and reference tube, while the ^19^F MR signal in the tumour was notably lower. This may be due to differences in NP distribution or local microenvironmental factors affecting MR signal decay. These findings highlight the potential of Gd multi-core NPs as a smart, pH-responsive probe capable of selectively activating ^19^F MRI contrast in acidic environments, making them advantageous for precision imaging and diagnosis in tumour microenvironments and other conditions where pH variations are critical.

Collectively, our results indicate that the pH responsiveness is not driven by Gd release, PLGA degradation, or specific ionic effects. At pH 3, reduced ^1^H *T*_1_ values (Table S2) suggest matrix swelling and increased permeability. However, at more physiological acidity (pH 5), ^1^H *T*_1_ remains comparable to pH 7 despite clear ^19^F signal enhancement, indicating that matrix softening alone is insufficient to explain the effect. Minor factors, such as localized PFCE mobility and other physicochemical changes, may contribute synergistically. Still, among all tested variables, internal ultrastructural rearrangement remains the most plausible and dominant mechanism driving the pH-dependent ^19^F MRI signal modulation.

Additionally, multi-core PFCE–PLGA NPs have previously demonstrated excellent biocompatibility, with no toxicity observed even at high doses, further supporting their suitability for clinical translation.^52^ Interestingly, previous studies with PFCE–PLGA NPs incorporating hydrophilic gadoteridol showed enhancement of the ^1^H MR signal, but no significant modulation of the ^19^F signal.^50^

Although our multi-core design leads to a lower absolute ^19^F signal and shorter *T*_2_ values (Fig. 2a, b and 3), it represents a rational design compromise, prioritizing stimulus-responsive behavior over maximal signal intensity. This approach is particularly well-suited for applications requiring conditional signal activation, such as imaging of pH-sensitive pathological environments.

This distinction is important: if the primary goal is to enhance the ^19^F signal under all conditions, other paramagnetic centers such as Fe^3+^ or Mn^2+^, which preferentially shorten *T*_1_ with minimal impact on *T*_2_, may be more appropriate.^35,54^ In contrast, our Gd-based multi-core system is designed to enable ^19^F ON/OFF switching. Here, effective modulation of the ^19^F signal depends not only on the presence of Gd chelates but also critically on maintaining a short Gd–^19^F distance, as achieved within the multi-core nanoparticle structure.

While the present *in vivo* experiments demonstrate proof-of-concept pH-responsive ^19^F MRI signal activation, intratumoral and subcutaneous administration do not fully reflect clinically relevant diagnostic scenarios, where intravenous delivery is currently the predominant route. Following systemic administration, dilution, clearance, and reduced local nanoparticle concentration are expected to further limit signal-to-noise ratio and detection sensitivity. In this context, the relatively low absolute ^19^F signal and short *T*_2_ values represent a limitation for whole-body diagnostic imaging. Nevertheless, by prioritizing conditional signal activation over maximal signal intensity, this platform may be particularly suited for applications involving localized accumulation, targeted delivery, or image-guided administration.

## 3 Conclusion

This study presents a novel pH-sensitive ^19^F MRI nanosystem in which NP ultrastructure modulates signal activation *via* the PRE effect, without requiring fluorophilic chelators or molecular linkers. By co-encapsulating PFCE and Gd-chelates within a PLGA matrix, we showed that multi-core NPs exhibit strong pH-responsive behaviour, enhancing ^19^F MR signal intensity under acidic conditions. In contrast, single-core NPs, representing the conventional structure for polymer-encapsulated PFCs, remained unaffected by pH changes. These findings were validated across high-field (14 T) and low-field (3 T) MRI platforms, as well as *in vitro* and *in vivo*, where tumour acidity selectively activated the ^19^F MR signal.

Notably, multi-core NPs exhibited reversible pH-dependent ^19^F signal enhancement under controlled *in situ* NMR conditions, a phenomenon not previously described in the literature. This reversibility supports the hypothesis that the observed effect is primarily driven by internal ultrastructural rearrangement, rather than by polymer degradation or Gd release, thereby preserving nanoparticle integrity. In contrast, single-core NPs remained pH-insensitive, emphasizing the role of nanoscale organization in modulating PRE-based contrast.

Given their high fluorine content, biocompatibility, and tunable response, Gd multi-core NPs offer a promising platform for stimulus-responsive MRI probes, with potential applications in tumour imaging and other pH-sensitive disease conditions. Their improved safety profile is further supported by the use of a biodegradable polymer matrix and chelated Gd, helping to mitigate concerns regarding free Gd toxicity. Moreover, the ability of these NPs to selectively activate the ^19^F signal only after cellular internalization provides an additional advantage for accurate and reliable ^19^F MRI-based cell tracking. More broadly, this work demonstrates that the nanoparticle architecture can be leveraged as a switchable contrast mechanism, offering a promising strategy for adaptive diagnostic imaging and stimulus-responsive theranostic applications.

## 4 Materials and methods

### 4.1 Materials

All chemicals were used as received. Poly(d,l-lactide-*co*-glycolide) (PLGA) Resomer RG 502H, with a lactide : glycolide molar ratio of 50 : 50, was obtained from Evonik Industries AG, Essen, Germany. Polyvinyl alcohol (PVA, 9000–10 000 *M*_w_, 80% hydrolyzed), dichloromethane (DCM), deuterium oxide (D_2_O), trifluoroacetic acid (TFA), gadolinium chloride (GdCl_3_), and 3-aminopropyl-triethoxysilane (APTES) were purchased from Sigma-Aldrich (St. Louis, MO, USA). Perfluoro-15-crown-5 ether (PFCE) was obtained from Exfluor (Texas, USA). Water was purified using a Purelab Chorus water purification system from Elga (Milli-Q water). Lipophilic gadolinium (Gd) chelate was synthesized as described previously.^67^ Phosphate-buffered saline (PBS), Dulbecco's Modified Eagle Medium (DMEM), fetal bovine serum (FBS), and 1% penicillin/streptomycin were purchased from Gibco, Thermo Fisher Scientific, USA.

### 4.2 Methods

#### 4.2.1. Synthesis of multi-core NPs

The detailed preparation of the NPs was described in our previous manuscripts.^50–52^ Briefly, PLGA (100 mg) was dissolved in DCM (3 mL), followed by the addition of PFCE (900 µL). Due to the immiscibility of PFCE in DCM, the mixture forms a two-phase system, which was continuously mixed by pipetting to ensure homogeneity. In parallel, a stock solution of Gd-chelate in DCM was prepared (*c* = 0.0166 mg µL^−1^), and the desired amount of Gd-chelate was added to the organic phase. This mixture was then added to 25 g of an aqueous solution of PVA (2 wt%) and emulsified in an ice-water bath using a sonicator (Sonifier 250; Branson Sonic Power, Danbury, CT, USA) at 40% amplitude for 3 minutes.

After sonication, the organic solvent was allowed to evaporate overnight, solidifying the particles. The NPs were washed four times with Milli-Q water by centrifugation at 16 000 × *g* (4 °C, 35 minutes) with supernatant replacement after each wash. The NPs were then resuspended in 5 mL of water, frozen using liquid nitrogen, and freeze-dried. The resulting powder was stored at −20 °C. Control NPs were prepared using the same procedure, excluding the addition of the Gd-chelate solution to the organic phase. For confocal microscopy experiments, NPs were prepared using the same procedure, with the addition of a fluorescent dye (AttoOxa12, ATTO-TEC GmbH, Germany) to enable imaging. In this manuscript, NPs containing Gd-chelate will be referred to as Gd multi-core NPs, while those without Gd-chelate will be referred to as control NPs (Fig. 1).

#### 4.2.2. Synthesis of single-core NPs

Single-core NPs were synthesized using an adapted multi-core NP synthesis protocol as previously described.^50,51^ A two-phase system consisting of PLGA (100 mg), DCM (3 mL), and PFCE (900 µL) was added to an aqueous sodium cholate solution (24 625 g, 1.5 wt%) and sonicated under the same conditions described above (40% amplitude, 3 minutes). Sodium cholate served as a surfactant during the sonication step. The desired amount of Gd-chelate (*c* = 0.0166 mg µL^−1^) was added to the organic phase prior to sonication. Following sonication, DCM was evaporated overnight under stirring to solidify the particles. To replace the surfactant, PVA solution (10 g, 2 wt%) was added to the emulsion, which was continuously stirred at 4 °C for 4 days. The single-core NPs were then washed four times with Milli-Q water through centrifugation (16 000 × *g*, 35 minutes, 4 °C), resuspended in 5 mL of water, frozen with liquid nitrogen, and freeze-dried. The resulting powder was stored at −20 °C. In this manuscript, single-core NPs containing Gd-chelate will be referred to as Gd single-core NPs (Fig. 1).

#### 4.2.3. Inductively coupled plasma optical emission spectroscopy (ICP-OES)

The Gd content in the NPs was determined using ICP-OES (Avio 500; PerkinElmer, Waltham, MA, USA) using GdCl_3_ and unloaded NPs as internal standards. Samples were prepared by adding 10 mg of the NPs to 2 mL mixture of nitric acid (HNO_3_, 69%) and hydrochloric acid (HCl, 37%) in a 3 : 1 volume ratio. The mixture was incubated at 40 °C for two days to ensure complete degradation of the NPs. After degradation, the samples were diluted to a total volume of 10 mL prior to measurement.

#### 4.2.4. Dynamic light scattering (DLS)

NPs at a concentration of 0.1 mg mL^−1^ were characterized for their diameter and polydispersity index (PDI) using DLS (Zetasizer Nano, Malvern Panalytical Ltd, UK). Measurements were carried out at a scattering angle of 173° using disposable cuvettes. The reported values represent the mean from four independent measurements, with each measurement comprising six runs of 10 seconds each, conducted at 25 °C.

#### 4.2.5. Atomic force microscopy (AFM)

Polymeric NPs were prepared for AFM analysis by depositing them onto positive functionalized mica substrates. The mica surface was first cleaved, and then functionalized by incubating it with 10 µL of APTES (0.1% v/v in Milli-Q water) for 1 minute. The substrate was then rinsed three times with 1 mL of Milli-Q water and dried using a gentle nitrogen stream. Subsequently, a solution of polymeric NPs (10 µL, 0.1 mg mL^−1^) at pH 3.0 and 7.0 was deposited onto the positive functionalized surface. The droplet was incubated for 10 minutes, rinsed with 1 mL of Milli-Q water, and dried with a gentle nitrogen stream. All preparation steps were conducted at room temperature under laminar flow. AFM maps of the 3D morphology were acquired for both samples in a regime of constant phase change,^68^ with 4 nm/pixel resolution by means of an NX10 (Park Systems, South Korea) operating in non-contact mode and equipped with an aluminum-coated cantilever (PPP-NCSTR, 7.4 N m^−1^) with a probe of nominal radius <7 nm. Mountains SPIP software (Digital Surf) and SPIP software (version 6.7.3, Image Metrology, Denmark) were used for image processing and further analysis. Cross-sectional profiles of individual particles under both conditions were extracted for statistical analysis (*n* = 76).

#### 4.2.6. Cell labelling

A murine macrophage cell line (RAW 264.7 cells, obtained from ATCC) was cultured in DMEM medium supplemented with 10% foetal bovine serum (FBS) and 1% penicillin/streptomycin under standard conditions (37 °C, 5% CO_2_). Cells were evaluated under two conditions: overnight incubation and premixing.

In the overnight incubation condition, cells were seeded in 6-well plates and incubated for ∼12 hours with Gd multi-core or control NPs at a concentration of 2 mg per 1.0 × 10^6^ cells to allow uptake. After incubation, excess NPs were removed by washing the cells three times with PBS, followed by scraping and resuspension in fresh medium.

In the premixing condition, cells were mixed with the desired NPs immediately before measurement, without allowing time for uptake.

For MRI measurements, the same conditions were used, except that after washing and collecting the cells, cells were fixed with 4% paraformaldehyde (PFA) at room temperature for 30 minutes. Following fixation, cells were washed three additional times, resuspended in PBS, and centrifuged to form a pellet before scanning.

#### 4.2.7. Fluorine-19 nuclear magnetic resonance ( ^19^F NMR)

^19^F NMR experiments were performed on a Bruker Avance III 400 MHz NMR spectrometer (Bruker, Ettlingen, Germany) equipped with a 5 mm BBFO+ probe at 298 K for quantitative analysis, or on a JEOL ECZ-R 500 MHz spectrometer (JEOL, Tokyo, Japan) equipped with a RoyalHFX probe to investigate pH effects on relaxation times in cells and various environments, including different organic acids. Measurements included ^19^F signal intensity, *T*_1_ (longitudinal), and *T*_2_ (transverse) relaxation times.

For quantitative ^19^F NMR spectroscopy, 5 mg of NPs was resuspended in 500 µL of D_2_O and mixed with 1 vol% TFA (100 µL) as an internal reference (*δ* = 0.00 MHz). The solution was transferred into a 5 mm NMR tube, and measurements were performed using 8 scans with a 25-second interscan relaxation delay.

For relaxation time measurements, 5 mg of NPs were resuspended in D_2_O or a D_2_O-based solvent with the desired pH at the same concentration used for quantitative analysis right prior to the measurement and transferred to an NMR tube. The ^19^F 90° pulse was calibrated prior to measurement and *T*_1_ and *T*_2_^19^F relaxation times were measured as described below.

*T*_1_ measurements were conducted using the inversion recovery method, which involves a 180° pulse followed by variable delay times to allow recovery of the longitudinal magnetization, followed by a 90° detection pulse. *T*_1_ was initially estimated using a simple 1D sequence, followed by a full pseudo-2D acquisition with delays and an interscan delay set to 5 × *T*_1_ (longest). Recovery curves were fit to an exponential growth function to calculate *T*_1_. The ^19^F *T*_1_ experiments were performed without ^1^H decoupling.

*T*_2_ measurements were performed using the Carr–Purcell–Meiboom–Gill (CPMG) sequence, which involves a 90° pulse followed by a train of tau-180-tau sequences, where tau is a variable delay. For rapidly *T*_2_-relaxing nuclei (*T*_2_ < 30 ms), a tau of 0.5 ms was used and for slower *T*_2_-relaxing nuclei, a tau of 1.2 ms was used. *T*_2_ was first estimated using a simple 1D sequence, followed by a full pseudo-2D acquisition with a list of refocusing pulse repetitions ranging from 2 to 1750 and an interscan delay set to 5 × *T*_1_ (longest). The recovery was fit to a mono-exponential decay function to determine *T*_2_ relaxation time.

For in-cell ^19^F NMR experiments cells were prepared as described above for the premixing and overnight incubation conditions. Following cell labelling, washing, and harvesting, cells were resuspended in 0.5 mL of full medium supplemented with 10% D_2_O and transferred to a 5 mm Shigemi tube. The inner Shigemi tube was omitted to prevent compression of the cells. Cell viability was assessed using trypan blue staining and quantified with an Invitrogen™ Countess™ 3 (Thermo Fisher Scientific, USA) automated cell counter. All NMR data analysis was performed using Mestrenova 14.3.0 (Mestrelab Research, Barcelona, Spain).

#### 4.2.8. Fluorine-19 magnetic resonance imaging (^19^F MRI)

##### 4.2.8.1. 14 T MRI

MR experiments with phantoms were conducted on a Bruker wide-bore 600 MHz Avance NEO spectrometer (Bruker, Ettlingen, Germany) equipped with a Micro 2.5 microimaging gradient system and Paravision 360 imaging software. A MicWB40 microMRI probehead, fitted with a 30 mm diameter dual-tuned ^1^H/^19^F coil, was used for all experiments. All ^19^F sequences were run with a chemical shift offset appropriate for the NP fluorine signal (approximately −91.6 ppm).

Fluorine and hydrogen MRI were performed on (1) Gd single-core NPs, (2) Gd multi-core NPs, and (3) control NPs at a concentration of 6.5 mg mL^−1^. Phantoms were prepared using 200 µL Eppendorf tubes, each containing NPs at pH 3, 5, or 7, along with a control tube containing water. All four tubes were placed in a 25 mm NMR tube for simultaneous scanning. To evaluate relaxation time changes following cell uptake, cells were treated as described in Section 4.2.6, using the overnight incubation and premixing conditions. *T*_1_ and *T*_2_ relaxation times were then measured for both conditions in the cell pellets and the supernatant.

*T*_1_ relaxation times were measured using a Flow-Sensitive Alternating Inversion Recovery with Rapid Acquisition with Relaxation Enhancement (FAIRRARE) sequence with the following parameters: echo time (TE) of 4.36 ms, repetition time (TR) of 5000 ms, and inversion times (TI) of 23, 100, 250, 500, 800, 2000, and 4000 ms, yielding seven MR images. The number of averages (NA) was set to 60 for the control NPs and 240 for the Gd NPs. The matrix size was 32 × 32, with a field of view (FOV) of 20 × 20 mm, a slice thickness (ST) of 10 mm, and a RARE factor of 32. Total scan time was 35 minutes for control NPs and 140 minutes for Gd NPs.

*T*_2_ relaxation times were measured using a multi-scan multi-echo (MSME) sequence with the following parameters: TE of 20 ms, echo spacing of 20 ms, and a total of 60 echo images. TR was 5000 ms, with NA set to 30. The matrix size was 32 × 32, with a FOV of 20 × 20 mm and a slice thickness of 10 mm. The total scan time was 80 minutes.

Phantom localization was established using a ^1^H RARE sequence with the following parameters: TE of 20 ms, TR of 1000 ms, NA of 4, matrix size of 256 × 256, FOV of 20 × 20 mm, slice thickness of 0.4 mm, and a RARE factor of 8. The scan time was approximately 2 minutes. A subsequent ^19^F RARE sequence was performed using the same orientation as ^1^H (axial). The parameters for the ^19^F MR sequence were: TE of 18 ms, TR of 2075 ms, NA of 512, matrix size of 40 × 34, FOV of 20 × 20 mm, slice thickness of 10 mm, and a RARE factor of 34, with a scan time of approximately 18 minutes.

##### 4.2.8.2 3 T MRI

To assess the pH sensitivity at clinically relevant magnetic field strengths, NPs were examined using a 3 T Bruker BioSpec system with ParaVision 360 v.3.3 software (Bruker, Ettlingen, Germany). The same conditions described in Section 4.2.8.1 were used, including the types of NPs, concentration, and phantom preparation.

*T*_1_ relaxation times were measured using an inversion recovery RARE sequence with a TE of 6.5 ms, a TR of 5000 ms, and TIs of 23, 100, 250, 500, 800, 2000, and 4000 ms, producing seven MR images. The NA was set to 60, with a matrix size of 32 × 32, a spatial resolution of 1.5 × 1.5 mm^2^ and a slice thickness of 5 mm. The RARE factor was set to 32, and the total scan time was 35 minutes.

*T*_2_ relaxation times were measured using a MSME sequence. Parameters for the control samples and the Gd-loaded sample at pH 3 were as follows: TE of 20 ms, echo spacing of 20 ms, and a total of 60 echo images, with a TR of 5000 ms and NA set to 60. The matrix size was 32 × 32, with a spatial resolution of 1.5 × 1.5 mm^2^ and a slice thickness of 5 mm. For the Gd samples at pH values of 5 and 7, the parameters were adjusted as follows: TE of 6.3 ms, echo spacing of 6.3 ms, and a total of 60 echo images, with a TR of 1800 ms and NA set to 60. The matrix size, spatial resolution and slice thickness remained the same.

#### 4.2.9. Confocal microscopy

Live cell fluorescence microscopy was performed under controlled conditions (37 °C, 5% CO_2_) with an inverted microscope Leica SP8 WLL (Leica Microsystems BV, Amsterdam, Netherlands) using a 63× oil-immersion lens. Images were acquired and post-processed with LAS X software (Leica Microsystems BV, Amsterdam, Netherlands). This experiment aimed to determine whether the ^19^F MRI signal observed after overnight incubation was due to the internalization of NPs into the acidic environment of lysosomes in RAW 264.7 cells. For this purpose, cells were seeded in 8-well chamber slides (LabTek, Nunc, Langenselbold, Germany) at a density of 5 × 10^3^ cells per well one day prior to the assay.

Fluorescently labelled multi-core NPs (AttoOxa12) were incubated at 2 mg per 1 × 10^6^ for either 12 or 8 hours prior to imaging. At each endpoint, cells were washed three times with PBS to remove excess NPs, and fresh medium with (or without) LysoTracker™ Green DND-26 (Thermo Fisher Scientific, USA) was added at a concentration of 100 nM for 2 hours. Fifteen minutes before washing, a drop of Hoechst EasyProbe (ABP Biosciences, Beltsville, USA) was added to each well for nuclear staining.

To image the premixing conditions, the particles were added to live cells *in situ* and immediately imaged. The acquisition settings were consistent between samples, and the same post-processing workflow was applied for the analysis.

#### 4.2.10. Proof-of-principle *in vivo* MRI (7 T MRI)

All animal procedures were conducted in accordance with ethical guidelines and approved by the Ethics Committee of the Institute for Clinical and Experimental Medicine and the Ministry of Health of the Czech Republic (approval no. 58/2014). The protocols adhered to the European Communities Council Directive (2010/63/EU) on the protection of animals used for scientific purposes. Adult Balb/c mice (6 months, *n* = 2, female) were housed in standard laboratory cages with a 12-hour light/dark cycle, in a conventional breeding facility, and were provided with water and pelleted food *ad libitum*. During injections of the NPs and MR experiments, anaesthesia was induced using 5% isoflurane (Baxter, Deerfield, USA) and maintained at 1.5 to 2%. Respiratory function was continuously monitored using a trigger unit (Rapid Biomedical, Berlin, Germany), and eye protection was ensured by applying eye cream (Ophthalmo-Septonex, Zentiva, Czech Republic). To maintain body temperature at 37 °C during MR measurements, animals were positioned in a custom coil holder equipped with water-filled tubes.

##### 4.2.10.1 4T1 cell incubation and tumor induction

The murine mammary carcinoma 4T1 cell line was cultured in Roswell Park Memorial Institute (RPMI) 1640 media (Gibco, Thermo Fisher Scientific) supplemented with 2 mmol per L l-glutamine, 10% FBS, and 1% penicillin–streptomycin. All cells were incubated under standard conditions (37 °C; 5% CO_2_). To induce tumour growth *in vivo*, a cell suspension (2 × 10^6^; *V* = 0.1 mL in PBS) was subcutaneously injected into the right mammary fat pad of healthy Balb/c mice. Tumour growth was monitored daily.

##### 4.2.10.2 NP phantom measurement

The NPs (control and Gd multi-core) were tested prior to the *in vivo* experiments using a custom-made dual ^1^H/^19^F surface radiofrequency (RF) coil on a 7 T MR experimental scanner (Bruker, Ettlingen, Germany). The half-saddle RF coil (30 mm diameter) was tuned and matched to the Larmor frequencies of both ^1^H and ^19^F nuclei for optimal performance. Phantom experiments of aqueous control NPs and Gd multi-core NPs (10 mg/100 µL) were performed to establish sensitivity. Phantom localization was achieved using ^1^H MRI with a *T*_2_-weighted turbo spin-echo RARE sequence with the following parameters: TR of 1000 ms, TE of 36 ms, 2 NA, RARE factor of 8, FOV of 20 × 20 mm^2^, matrix size of 256 × 256 and 0.078 × 0.078 mm^2^ resolution. ^19^F MR non-localized spectroscopy (^19^F MRS) was assessed using a single-pulse sequence with a TR of 1000 ms, a bandwidth (BW) of 41 ppm, and a NA of 1–600 to observe the MR sensitivity at different scan times (1 second to 10 minutes). The chemical offset (−91 ppm) was determined from the phantom measurements. ^19^F MR images were acquired by MR spectroscopic imaging (MRSI), which enables spectrally resolved signal acquisition from a 2D voxel slab, providing targeted signal separation at a specific resonance frequency. Following parameters were used: TR of 30 ms, BW of 80 ppm, FOV of 20 × 20 mm^2^, total scan time of 30 seconds to 1 hour (NA of 1–175), slice thickness of 5 mm, matrix size of 64 × 64 and 0.313 × 0.313 mm^2^ resolution. All phantom images were measured in the coronal plane.

##### 4.2.10.3 Tumour imaging

*In vivo* ^19^F MRS measurements were performed using the same RF dual coil and MR sequence parameters on a 7 T MR scanner as in phantom experiments, with a modified BW (50 ppm). The ^19^F MRSI sequence (TR of 100 ms) was optimized for the animal measurements with a FOV of 35 × 35 mm^2^, a slice thickness of 25 mm, and a scan time of 10 minutes (NA of 17). Measurements were conducted directly after NP injection (5 mg/100 µL), administered subcutaneously (SC) and intratumorally (IT), at 24 hours post-injection (day 1) and on day 5 (only control NPs). Control NPs and Gd multi-core NPs (2 mg/100 µL) were mixed in 4% gelatine and used as signal references placed next to the animal. Tumour containing Gd-multi-core NPs was extracted at day 1 and measured *ex vivo* using the same sequences.

## Conflicts of interest

There are no conflicts of interest to declare.

## Supplementary Material

NA-008-D5NA01005E-s001

## Data Availability

All data supporting the findings of this study are available within the article and its supplementary information (SI). Supplementary information: Fig. S1–S5 and Tables S1–S7 providing additional data supporting the main findings of the manuscript. These include detailed nanoparticle size and polydispersity measurements by DLS, ^19^F and ^1^H MR relaxometry data, reversibility experiments confirming pH sensitivity, and MR images of *in vitro* and *ex vivo* samples. See DOI: https://doi.org/10.1039/d5na01005e.
