# Supplementary material for: Nanoparticle ultrastructure allows reversible pH sensitivity using 19F NMR and in vivo19F MRI
Source: Nanoscale Adv. 2026 Jan 28;8(6):1975–91. doi: 10.1039/d5na01005e (PMC12915682; doi:10.1039/d5na01005e)
Supplement: NA-008-D5NA01005E-s001 [file NA-008-D5NA01005E-s001.pdf]

Supplementary Information (SI) for Nanoscale Advances.
This journal is © The Royal Society of Chemistry 2026

Supporting Information to

Nanoparticle ultrastructure allows reversible pH sensitivity using ^{19}F NMR and in vivo ^{19}F MRI

Alvja Mali[1], Mariah Daal[1], Natalia Jirát-Ziółkowska[2,3], Nicolas Stumpe[4], Naiara Larreina Vicente[1], N. Koen van Riessen[1], Visakh V. S. Pillai[5], Francesco Simone Ruggeri[5], Cyril Cadiou[6], Françoise Chuburu[6], Daniel Jirak[2,7], Paul B. White[8], Mangala Srinivas[1,9,*].

[1]Department of Cell Biology and Immunology, Wageningen University and Research, Wageningen, The Netherlands.
[2]Institute for Clinical and Experimental Medicine, Prague, Czech Republic.
[3]Institute of Biophysics and Informatics, First Faculty of Medicine, Charles University, Prague, Czech Republic.
[4]Institute for Molecular Cardiology, Heinrich Heine University, Düsseldorf, Germany.
[5]Physical Chemistry and Soft Matter, Wageningen University and Research, Wageningen, The Netherlands.
[6]University of Reims Champagne Ardenne, CNRS, ICMR UMR 7312, Reims, France.
[7]Faculty of Health Studies, Technical University of Liberec, Liberec, Czech Republic
[8]Institute for Molecules and Materials, Radboud University, Nijmegen, The Netherlands.
[9]Cenya Imaging B.V., Amsterdam, The Netherlands.

***Corresponding authors:**
mangala.srinivas@wur.nl
Wageningen University & Research
Cell Biology and Immunology
P.O. Box 338, 6700 AH Wageningen, The Netherlands

NPs	Size (nm)	PDI
Gd multi-core	200 ± 20	0.2
Gd single-core	160 ± 10	0.15
Control	200 ± 25	0.2

Table S1. Average size and PDI of NPs measured by DLS. Data are presented for three types of NPs: Gd multi-core, Gd single-core, and control. The values represent the average diameter (nm) and PDI, based on measurements from 9, 4, and 5 batches, respectively, used in this study.

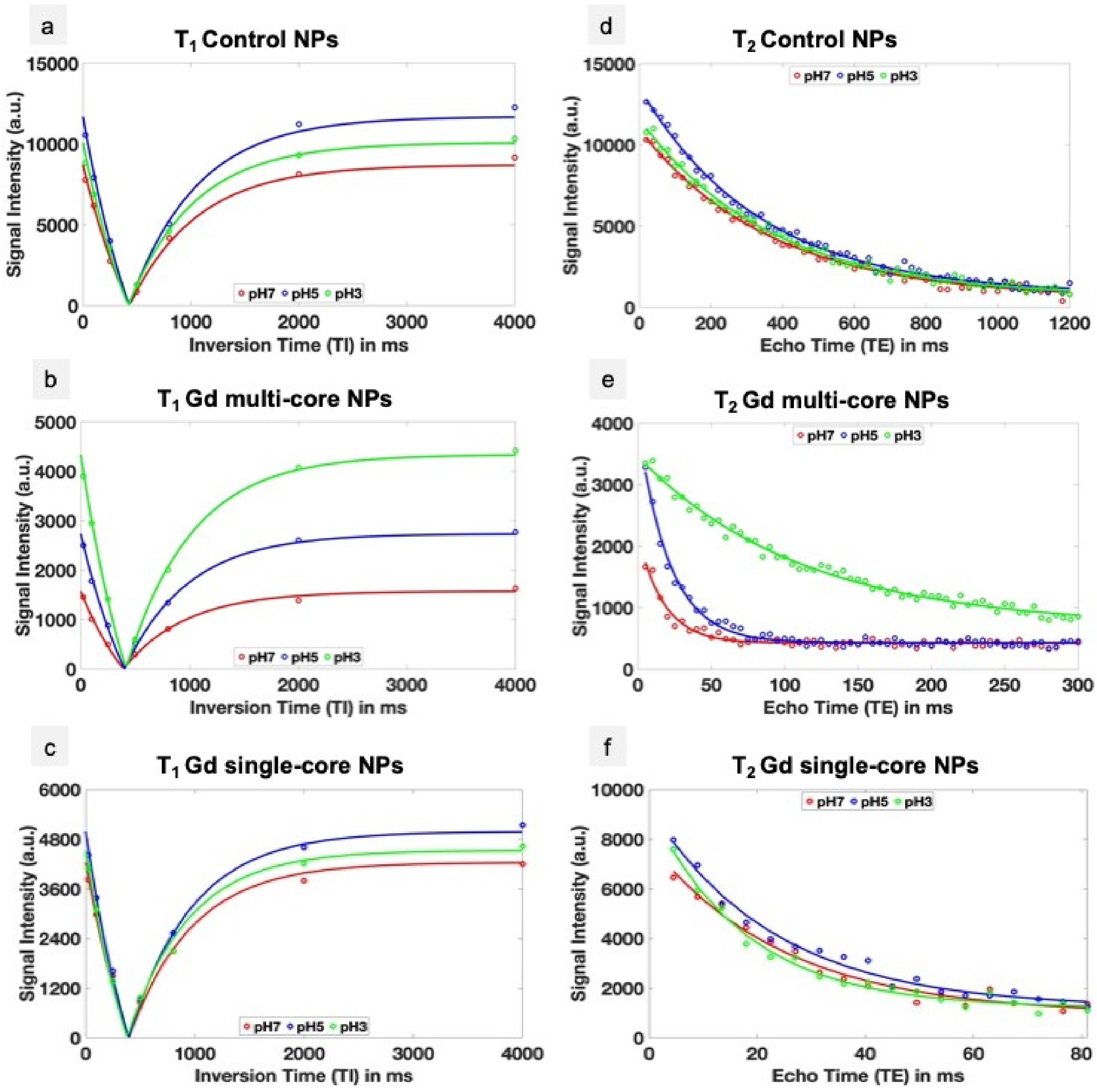

Figure S1. ^{19}F MR T_1 and T_2 relaxation times curves measured at 14 T for control (a,d), Gd multi-core (b,e) and Gd Single-core (c,f) NPs.

pH	$^{1}H\ T_1$ (ms)
7	1248
5	1260
3	1080
H_2O	1672

Table S2. $^{1}H\ T_1$ relaxation times (ms) measured by 14 T MR for Gd multi-core NPs at pH 7, 5, and 3. Water was used as a reference.

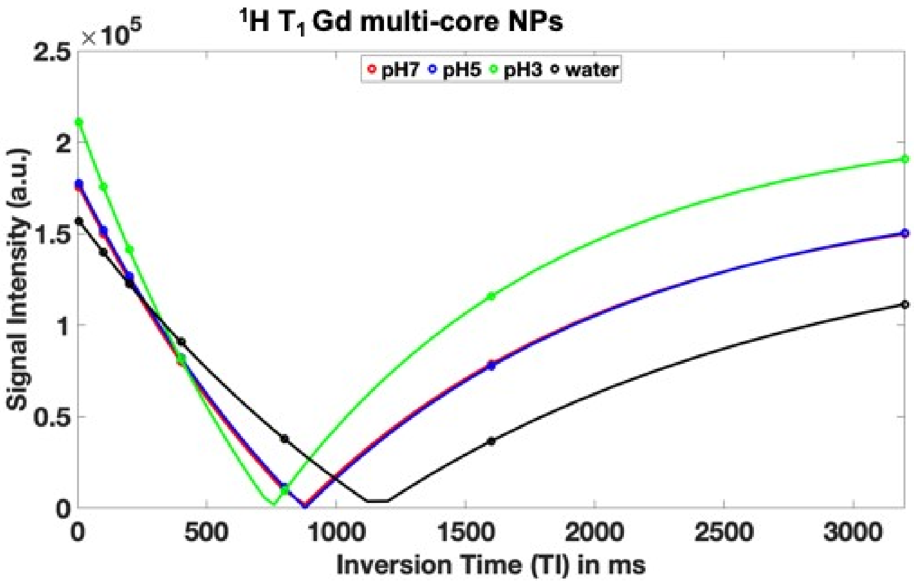

Figure S2. $^{1}H\ T_1$ curve measured at 14 T MR for Gd multi-core NPs at pH 7, 5, and 3. Water was used as the control

pH	Solvent	Size (nm)	PDI
7	D_2O	228 ± 0.5	0.1
3	Formic Acid	230 ± 0.5	0.1
3	Acetic Acid	231± 2.5	0.06
3	HCl	229± 1.5	0.07

Table S3. DLS measurements (diameter and PDI) of Gd multi-core NPs after ^{19}F NMR analysis. The NPs were resuspended in deuterium oxide (D_2O) as a control (pH 7) and in various organic acids (pH 3): formic acid, acetic acid, chloridric acid (HCl).

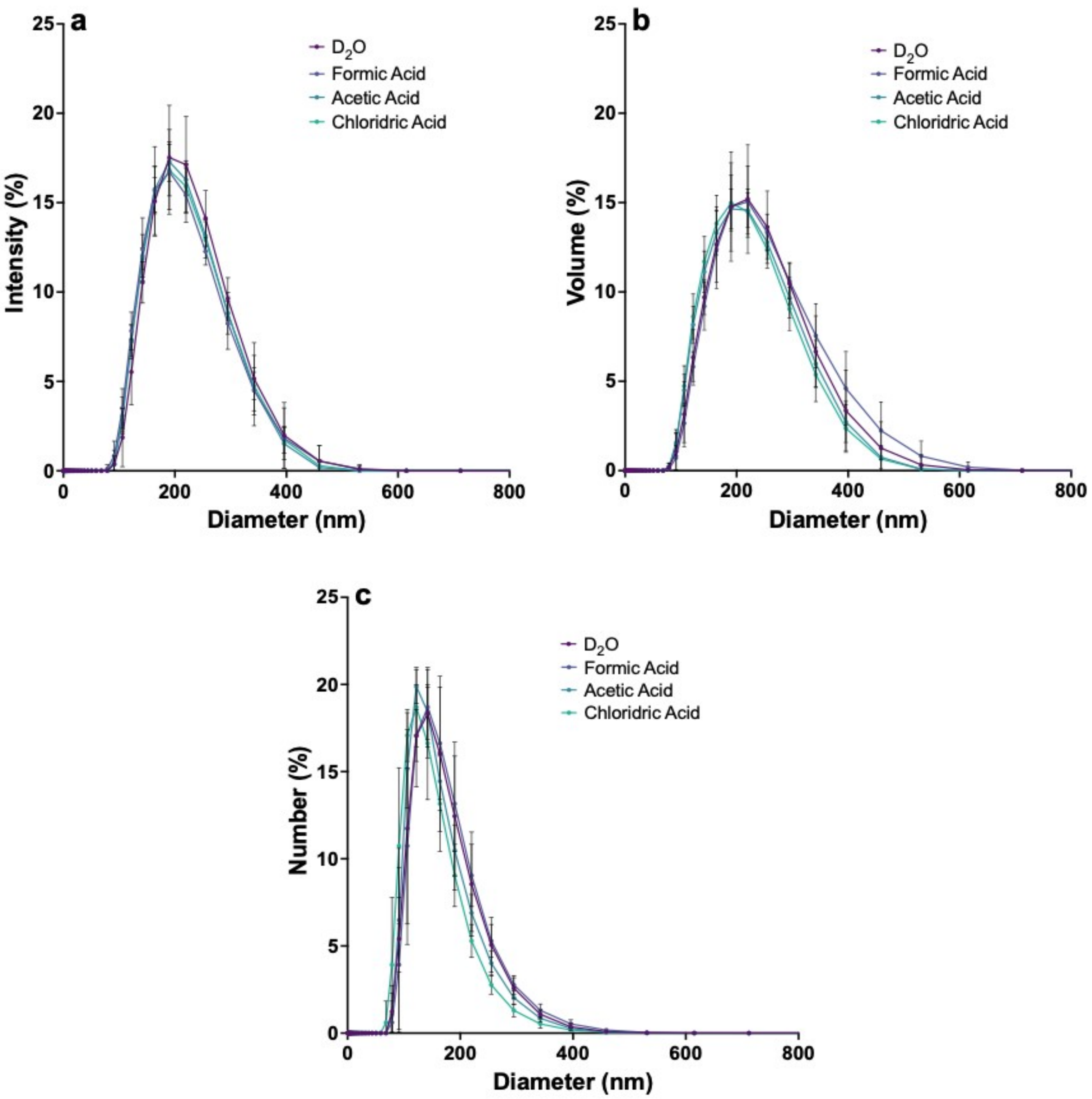

Figure S3. Size distribution of Gd multi-core NPs after ^{19}F NMR analysis, presented as (a) intensity, (b) volume, and (c) number. The NPs were resuspended in various organic acids (formic acid, acetic acid, hydrochloric acid and deuterium oxide (D_2O)) as a control. The graphs represent the average of four independent measurements, including standard deviations.

pH	Size (nm)	PDI
3	194 ± 4	0.06
7	192 ± 2	0.06
7 from pH 3	194 ± 3	0.07

Table S4. DLS measurements (diameter and PDI) of Gd multi-core NPs after the ^{19}F NMR reversibility experiment. Three samples from the same batch were analysed: one resuspended at pH 3, one at pH 7, and another that was initially resuspended at pH 3 and then neutralized to pH 7 using 5N NaOH.

pH	T_2 (Batch_1)	T_2 (Batch_2)
3	24	17
7	162	189
7 from pH 3	28	15

Table S5. Additional batches for reversibility experiments. ^{19}F T_2 relaxation times measured for two independently prepared Gd multi-core nanoparticle batches (Batch 1 and Batch 2) resuspended at pH 7, pH 3 (HCl solution), and after neutralization back to pH 7 (“7 from pH 3”) using 5 N NaOH. All measurements were performed by ^{19}F NMR at 500 MHz under identical experimental conditions, with a nanoparticle concentration of 6.5 mg/mL.

pH	^{19}F T_2(ms)
7	15
3	160
7 from pH 3	18

Table S6. ^{19}F T_2 values of Gd multi-core NPs resuspended in acetic acid at pH 7, pH 3, and initially at pH 3 and then neutralized to pH 7 using 5N NaOH. Measurements were performed using ^{19}F NMR at 500 MHz.

Control NPs	^{19}F T_2 (ms)
Overnight uptake	759
Premixing	780
NPs	750

Gd Multi-core NPs	^{19}F T_2 (ms)
Overnight uptake	208
Premixing	30
NPs	28

Table S7: ^{19}F T_2 values of Gd multi-core NPs and control NPs at time 0 (premixing), after 12 hours (overnight uptake), and for NPs alone. Measurements were performed at 37°C using ^{19}F NMR at 500 MHz.

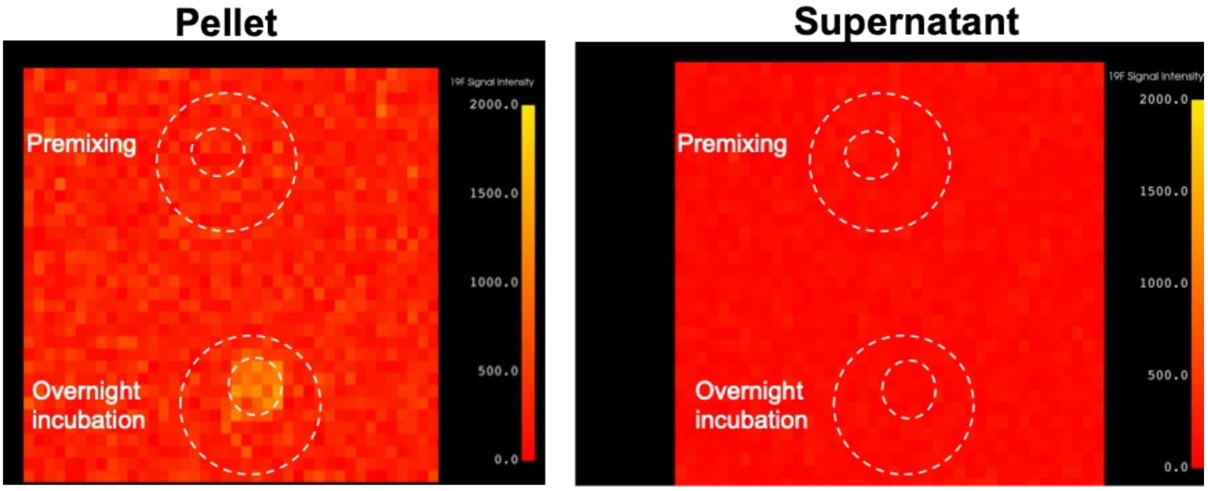

Figure S4: ^{19}F MR images (14T) of the RAW 264.7 cells pellet and supernatant after premixing and overnight incubation with Gd multi-core NPs without thresholding.

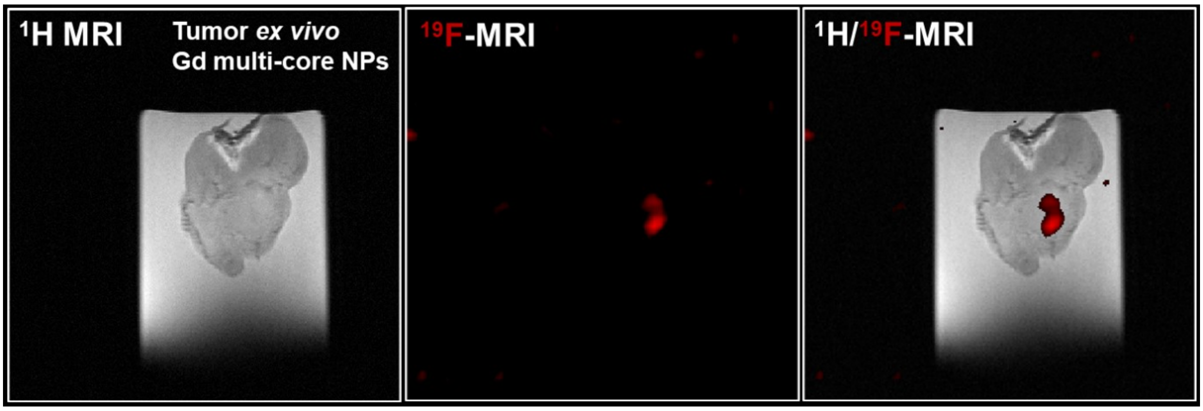

Figure S5. ^{1}H and ^{19}F MR images (7T) of the tumour *ex vivo.* The ^{19}F MR signal confirm the Gd multi-core NPs (IT injection) responsiveness in lower pH of the tumor microenvironment.
